# Prevalence of Intestinal Parasitic Infections and Associated Factors Among Rural Ghanaian School Children: A Cross‐Sectional Study in Tokuroano, Krachi East Municipality

**DOI:** 10.1002/puh2.70176

**Published:** 2026-01-29

**Authors:** Christopher Yaw Dumevi, Prince Wise Amekudi, Nana Aba Setorwu Eyeson, Hugette Naa Ayele Aryee, Joyce Junior Asiamah, Ezekiel Kofi Vicar, James‐Paul Kretchy, Simon Sovoe, Saviour Kweku Adjenti, Nicholas T. K. D. Dayie, George Boateng Kyei, Patience B. Tetteh‐Quarcoo, Irene Ayi, Patrick F. Ayeh‐Kumi

**Affiliations:** ^1^ Department of Physician Assistantship Studies School of Medical Sciences Central University Accra Ghana; ^2^ Department of Medical Microbiology University of Ghana Medical School Accra Ghana; ^3^ Department of Public Health School of Medical Sciences Central University Accra Ghana; ^4^ Department of Clinical Microbiology School of Medicine University for Development Studies Tamale Ghana; ^5^ Department of Research Technology and Innovation Environmental Protection Authority Accra Ghana; ^6^ Department of Community Health Ensign Global University Akosombo Ghana; ^7^ Department of Virology Noguchi Memorial Institute for Medical Research College of Health Sciences University of Ghana Accra Ghana; ^8^ Department of Parasitology Noguchi Memorial Institute for Medical Research College of Health Sciences University of Ghana Accra Ghana

**Keywords:** child health, hygiene, intestinal parasites, mono parasitism, polyparasitism

## Abstract

**Background:**

Intestinal parasitic infections (IPIs) pose a significant public health burden in developing regions, disproportionately affecting children. This study investigated the prevalence, determinants, and co‐occurrence patterns of IPIs among schoolchildren in rural Krachi East Municipality, Oti Region, Ghana.

**Methodology:**

A cross‐sectional study (March–August 2024) enrolled 204 schoolchildren (aged 4–15 years) via simple random sampling. Sociodemographic and risk factor data were collected, using standardized questionnaires. Single fresh stool specimens were microscopically examined using direct wet mount, sedimentation, and Kato–Katz techniques. Data were analyzed with STATA version 18.

**Results:**

Overall IPI prevalence was 63.23% (*n* = 129), with monoparasitism at 33.3% (*n* = 68). Key parasites included *Ascaris lumbricoides* (57.4%), Hookworm (38.7%), and *Giardia duodenalis* (21.6%), *Entamoeba dispar/histolytica* (14.2%), and *Trichuris trichiura* (11.3%). Polyparasitism was common, with protozoan–helminth (19.38%) and helminth–helminth (27.13%) co‐infections. *A. lumbricoides* and Hookworm were predominant in co‐infections. Home‐cooked meals and awareness of IPIs reduced the odds of infection. Conversely, Kumasi Ventilated Improved Pit (KVIP) and pit latrines at school paradoxically increased *G. duodenalis* infection risk, whereas fingernail‐biting showed a counterintuitive protective association against *G. duodenalis* infections.

**Conclusion:**

A high IPI burden, including significant polyparasitism, exists among the schoolchildren in the study area. The findings underscore the urgent need for integrated deworming programs, improved sanitation, and targeted health education to reduce morbidity in this vulnerable population, directly contributing to SDG 3 (Good Health and Well‐being) by addressing preventable diseases and promoting health, and SDG 4 (Quality Education) by improving the health and educational outcomes of schoolchildren.

## Graphical abstract

Over 63% of schoolchildren in rural Ghana's Tokuroano area had intestinal parasitic infections, mainly *Ascaris*, Hookworm, and *Giardia*. Poor sanitation, unsafe water, and poor hygiene were key risks. Protective factors included home‐cooked meals and health education. Integrated deworming, sanitation, and education programs are urgently needed to address this public health issue.

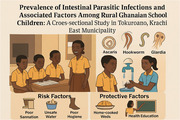

## 1 Introduction

Intestinal parasitic infections (IPIs) are distributed worldwide [1], and are significant global public health challenge, particularly in low‐ and middle‐income countries (LMICs), where environmental and socio‐behavioral factors facilitate transmission [2]. An estimated 3.5 billion people are affected by intestinal parasites, resulting in over 450 million symptomatic cases and approximately 200,000 deaths annually worldwide [3, 4], with children disproportionately affected [5, 6].

Despite global deworming efforts, IPIs remain a significant health burden in sub‐Saharan Africa, particularly among children with varying prevalence rates reported; 33% in Ethiopia [7], 23.6% [8], 23.95% [9], 86.2% [10] in Nigeria, 84% in Colombia [11], 71.4% in Malaysia [12], 10.7%–33% in Kenya [13], and 64.4% in Sudan [14], highlighting persistent transmission in high‐risk rural settings. In Ghana, IPIs remain endemic despite mass drug administration (MDA) campaigns and improved sanitation, with prevalence rates of 19.5% [15] and 26.79% [16] in Accra, and 12.5% in Elmina, in the Central region [17]. The IPI prevalence in the Volta Region ranges between 14% [18] and 35.1% [19] in the Ho Municipality, whereas 44.08% prevalence was reported in the Kadjebi District [20] of the Oti Region.

It is worth noting that most studies in Ghana have focused on urban or peri‐urban areas and single‐pathogen investigations, overlooking broader epidemiological patterns and the complexities of co‐infections (polyparasitism) in rural parasite ecology. Polyparasitism, the simultaneous infection with two or more intestinal parasite species, significantly contributes to the diarrheal disease burden, especially among children in rural communities, due to their underdeveloped immunity and poor hygiene behaviors [11, 12]. Affected children often present with symptoms, such as nausea, stomach cramps, vomiting, diarrhea, malnutrition, stunted growth, and fever, which, without timely medical intervention, can severely impair their health and educational outcomes [21].

Children in rural settings face increased exposure to contaminated water, soil, food, and surfaces due to inadequate sanitation, limited access to safe water, and behaviors such as barefoot walking, geophagia, and nail biting [19, 22, 23]. This cumulative exposure heightens their susceptibility to both single infections and complex co‐infections, complicating diagnostic, treatment, and control strategies. Although IPIs are preventable and manageable, limited education and pervasive poverty leave vulnerable populations at considerable risk of associated debilitating conditions. Despite ongoing efforts to control soil‐transmitted helminth (STH) infections through preventive chemotherapy, water, sanitation, and hygiene (WASH) interventions, and community‐led total sanitation (CLTS) initiatives, infection persists often due to inadequate case management within primary healthcare facilities [24, 25].

School‐aged children (5–15 years) represent a key population for monitoring community‐level parasitic burden due to their high exposure risk and critical role in public health control programs. Nevertheless, this group remains under‐investigated regarding localized environmental factors, sanitation infrastructure, and cultural behaviors affecting disease transmission. In Ghana, studies have demonstrated that parental education, access to toilets, water sources, and hygiene practices are linked to specific parasite species or coinfection profiles [19, 26, 27]. Hence, understanding these associations is crucial for developing targeted public health interventions.

This study therefore aimed at providing crucial epidemiological data on IPI prevalence, co‐occurrence patterns, and associated sociodemographic and behavioral factors among schoolchildren in Tokuroano, a rural community in Ghana's Krachi East Municipality. This localized focus allows for the assessment of residual transmission despite national deworming efforts and facilitates the identification of persistent, modifiable risk factors, which is essential for implementing methods.

## 2 Methods

### 2.1 Study Design, Population, and Procedure

A cross‐sectional survey was conducted on 204 school children aged 4–15 years in Tokuroano D/A Primary School in the Krachi East Municipality, Oti Region, Ghana from March to August 2024. Simple random sampling technique was used to select the school and the study participants.

### 2.2 Questionnaire Administration

A standardized questionnaire was administered in English, and the local language (Twi and Ewe) to collect demographic and socioeconomic data from the study participants such as age, parents’ educational level, parents’ occupation, awareness of IPIs, source of water, source of food, type of toilet facility, as well as risk factors associated with the infection as described by Athiyyah et al. [28].

### 2.3 Study Site

**FIGURE 1:**
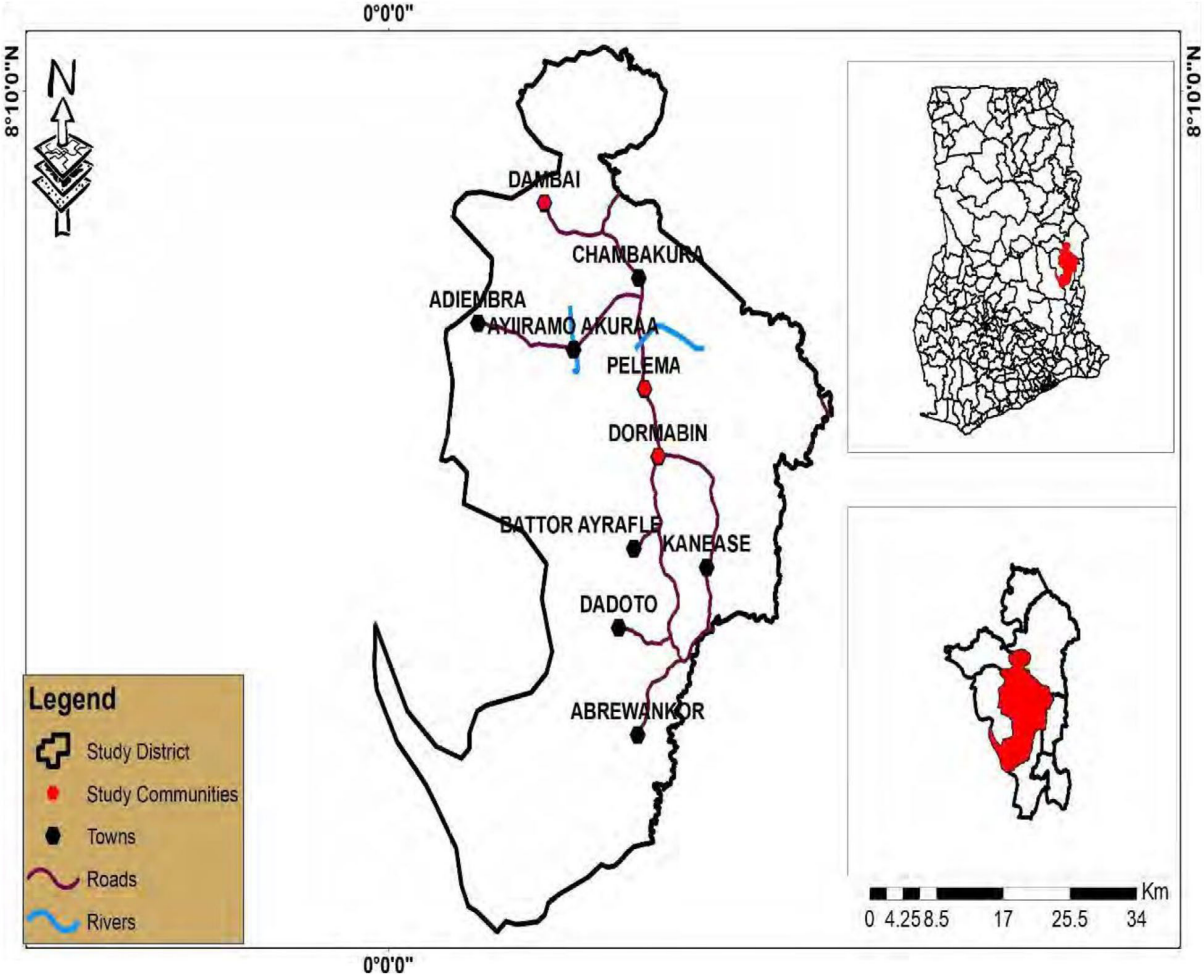
Map of the Krachi East Municipality, Oti Region [31].

The study was conducted at Tokuroano D/A Primary School, in Krachi East Municipality of the Oti Region. The Krachi East Municipality is one of the 261 Metropolitan, Municipal, and District Assemblies (MMDAs) in Ghana and is part of the eight municipalities and districts in the Oti Region. It is located in the northwestern part of the Volta Region, situated between latitudes 7°40′ N and 8°15′ N and longitudes 0°6′ E and 0°20′ E. It has Dambai as its administrative capital and the total area of the municipality is 2298 km^2^ of which approximately 15% is fresh water. The Krachi East Municipality shares its boundaries with Krachi West to the north, Biakoye District to the south, the Volta Lake to the west, and Kadjebi District to the east [29]. The total population of the Krachi East Municipality based on the 2021 Population and Housing Census is 110,435, comprising 56,186 males and 54,249 females with more higher rural population (75,385) compared to the urban dwellers (32,625) [30] (Figure 1).

### 2.4 Local Ecosystem and Burden of IPIs

The local ecosystem of Tokuroano in the Krachi East Municipality of the Oti Region impacts the spread of IPIs. Open defecation and inadequate improved sanitation facilities increase environmental fecal contamination risks. The contamination of communal streams facilitates the spread of parasite ova and cysts. Drinking water sources, such as boreholes, pipe‐borne water, sachet water, and sometimes river/stream water, pose exposure risks, especially for children. The high annual rainfall and humidity create optimal conditions for the survival of some intestinal parasites, whereas uncontrolled animal grazing contaminates the environment with zoonotic parasites, establishing a reservoir for human infection. This combination of environmental factors, inadequate sanitation, and unsafe water sources perpetuates the burden of parasitic diseases in the community.

### 2.5 Sample Size Determination

The sample size, *n*, was calculated using the Cochran formula *n = *(*Z* ^2^ *× p*(1* − p*))/*e* ^2^ [32]. Given a 95% confidence interval (CI), the standard score (*Z*) of 1.96, 5% margin of error (*e*), estimated prevalence (*p*) of 15% on the basis of similar study conducted by Forson et al. [33], *n* = (1.96)^2^ × 0.15(1 − 0.15)/(0.05)^2^. Hence, the minimum sample size *n = *0.490004/0.0025 = 196.00.

To account for potential contingencies such as non‐response or data attrition, a 5% contingency was added, resulting in a final adjusted sample size of approximately 206.

### 2.6 Stool Specimen Collection

Study participants received clear, illustrated instructions on proper stool collection, including urinating prior to defecation to avoid urine contamination. Each child was provided with a clean, dry, wide‐mouthed, screw‐capped, leak‐proof container fitted with a scoop and labeled with a unique specimen ID, date, and location. The container design minimized contact with toilet water or surfaces during defecation. Trained volunteer teachers supervised the process to ensure adherence to collection protocols and immediate handling of samples. Participants were instructed to fill the container approximately one‐third with fresh stool. Single stool specimens were collected privately in school toilet facilities and promptly transported for laboratory analysis.

To reduce the risk of possible infections, thorough handwashing with antiseptic soap and running water was performed by pupils and volunteer female teachers who assisted the younger children. A total of 20 stool specimens were collected from each pupil, and younger children were assisted during the specimen collection by volunteer female teachers. The stool specimens were fixed with 10% formalin [28] and transported to Anfoega Catholic Hospital for laboratory examination. Samples were collected between 8:30 a.m. and 9:30 a.m. each school day, and the laboratory processing and examination were conducted between 10:30 a.m. and 4:00 p.m. daily.

### 2.7 Laboratory Investigation

Stool specimen consistency was observed and categorized as formed, semi‐formed, loose, mucoid, slimy, or watery. A parasitological examination was conducted to evaluate the presence of ova, larvae, trophozoites, or cysts of helminths (*Ascaris lumbricoides*, Hookworm, *Trichuris trichiura*) and protozoa (*Giardia duodenalis*, *Entamoeba dispar/histolytica*). Positive infections were confirmed by detecting one or more parasites on prepared slides. Mono‐parasitic infection was defined as the presence of a single protozoan or helminthic parasite. Poly‐parasitic infection was defined as (1) ≥2 positive protozoan parasites; (2) ≥2 helminthic parasites; or (3) a mixed infection involving ≥1 protozoan and ≥1 helminthic parasite [28]. Stool samples were processed using standard parasitological techniques. Direct saline wet mount and iodine preparations were employed for identifying protozoan trophozoites and cysts. The formalin–ethyl acetate sedimentation technique was utilized to concentrate protozoan cysts and helminth eggs and larvae, particularly for low‐intensity infections [19]. The Kato–Katz method, using a 41.7 mg template, detected and quantified helminth eggs, specifically those of *A. lumbricoides, T. trichiura*, and hookworm species [34]. Both low‐ (10×) and high‐power (40×) objective lenses were used for examining stool specimens for enteric parasites. Three microscopists analyzed the slides: two independently, with a third resolving discordant results. The high‐power (40×) objective was specifically used to observe morphological features of motile organisms, including protozoan trophozoites or larvae.

### 2.8 Data Analysis

Frequencies, percentages were used for the variables of age, parent's occupation, toilet facility, main source of water, hygienic practices, and polyparasitic infections. Bar charts and Heat maps were performed to demonstrate the distribution of the mono and polyparasitic infections. Chi‐square test was used to determine the association between parasitic infections and socioeconomic variables. Multivariate logistic regression analysis was performed to evaluate paired associations between risk factors, such as gender, parents’ occupation, awareness of IPIs, type of toilet at home, type of toilet at school, source of food at school, and fingernails biting/thumb sucking, parasitic infections. All statistical analyses were performed with 95% CI, and the differences were set significant at a significance level of 5% (*p* ≤ 0.05). Data were analyzed using Excel and STATA version 18.

## 3 Results

Table 1 highlights the sociodemographic features of the 204 school children in the study with a median age of 7 (5–9, IQR). The study included 131 boys (64.22%) and 73 girls (35.78%). The majority of the parents attained basic education; 94 father (64.08%) and 98 mother (48.04%) with farming being the predominant occupation, 114 (55.88%) and 126 (61.76%) for fathers and mothers, respectively. The majority of the children who bought food from the street or at school was 114 (55.88%), whereas open defecation was commonly practiced, 25 (12.25%). Nearly half of the children wash their hands sometimes with soap and bite their fingernails or suck their thumbs. Stomach pain, 66 (32.4%) and diarrhea, 59 (28.9%) were the common symptoms the children frequently reported.

**TABLE 1:** The characteristic features of school children in the study.

Characteristic	*Ascaris lumbricoides*		*Giardia duodenalis*		*Entamoeba dispar/histolytica*		*Trichuris trichiura*		Hookworms		*N* = 204 (%)
	(*n* = 117)	*p* value	(*n* = 44)	*p* value	(*n* = 29)	*p* value	(*n* = 23)	*p* value	(*n* = 79)	*p* value	
**Age**											
(Median, IQR)	7 (6–10)		6 (5–9)		7 (6–9)		6 (5–8)		6 (5–9)		7 (5–9)
**Gender**											
Female	45 (38.46)	0.22	10 (22.73)	0.03	12 (41.38)	0.32	10 (43.48)	0.28	22 (27.85)	0.04	73 (35.8)
Male	72 (61.54)		34 (77.27)		17 (58.62)		13 (56.52)		57 (72.15)		131 (64.2)
**Parents educational level**											
No formal education	6 (5.13)	0.75	4 (9.09)	0.35	1 (3.45)	0.38	1 (4.35)	0.17	6 (7.59)	0.38	13 (6.4)
Basic education	55 (47.01)		23 (52.27)		10 (34.48)		7 (30.43)		31 (39.24)		94 (46.1)
Secondary education	51 (43.59)		17 (38.64)		17 (58.62)		13 (56.52)		40 (50.63)		90 (44.1)
Tertiary education	5 (4.27)		0		1 (3.45)		2 (8.70)		2 (2.53)		7 (3.4)
**Parents occupation**											
Civil servant	12 (10.26)	0.39	4 (9.09)	0.43	3 (10.34)	0.07	2 (8.70)	0.64	13 (16.46)	0.54	25 (12.3)
Farmer	66 (56.41)		23 (52.27)		12 (41.38)		14 (60.87)		42 (53.16)		114 (55.9)
Trader	35 (29.91)		13 (29.55)		10 (34.48)		5 (21.74)		20 (25.32)		55(26.9)
Unemployed	4 (3.42)		4 (9.09)		4 (13.79)		2 (8.70)		4 (5.06)		10(4.9)
**Awareness of intestinal parasitic infection**											
Yes	84 (71.79)	0.31	34 (77.27)	0.33	16 (55.17)	0.02	13 (56.52)	0.05	56 (70.89)	0.30	150 (73.5)
No	33 (28.21)		10 (22.73)		13 (44.83)		10 (43.48)		23 (29.11)		54 (26.5)
**Often become unwell**											
Every 4 months	13 (11.11)	0.92	5 (11.36)	0.72	2 (6.90)	0.18	5 (21.74)	0.23	10 (12.66)	0.94	24 (11.8)
Every 8 months	57 (48.72)		24 (54.55)		19 (65.52)		9 (39.13)		38 (48.10)		100 (49.0)
Every 12 months	47 (40.17)		15 (34.09)		8 (27.59)		9 (39.13)		31 (39.24)		80 (39.2)
**Type of toilet facility at home**											
Open defecation	16 (13.68)	0.20	5 (11.36)	0.30	7 (24.14)	0.13	3 (13.04)	0.85	6 (7.59)	0.27	25 (12.3)
Public toilet	26 (22.22)		8 (18.18)		7 (24.14)		7 (30.43)		22 (27.85)		55 (26,9)
Within house	75 (64.10)		31 (70.45)		15 (51.72)		13 (56.52)		51 (64.56)		124 (60.8)
**Type of toilet facility at school**											
Open defecation	15 (12.82)	0.17	1 (2.27)	0.03	8 (27.59)	0.06	7 (30.43)	0.13	11 (13.92)	0.69	30 (14.7)
KVIP	35 (29.91)		13 (29.55)		4 (13.79)		3 (13.04)		23 (29.11)		50 (24.5)
Pit latrine	37 (31.62)		18 (40.91)		6 (20.69)		6 (26.09)		24 (30.38)		65 (31.9)
WC	30 (25.64)		12 (27.27)		11 (37.93)		7 (30.43)		21 (26.58)		59 (28.9)
**Main source of drinking water at home**											
Borehole/well	31 (26.50)	0.86	5 (11.36)	0.10	10 (34.48)	0.48	3 (13.04)	0.35	23 (29.11)	0.65	50 (24.5)
Pipe‐borne water	38 (32.48)		17 (38.64)		7 (24.14)		7 (30.43)		24 (30.38)		69 (33.8)
River/stream	33 (28.21)		17 (38.64)		8 (27.59)		10 (43.48)		23 (29.11)		60 (29.4)
Sachet water	15 (12.82)		5 (11.36)		4 (13.79)		3 (13.04)		9 (11.39)		25 (12.3)
**Source of food while at school**											
Buy from the street/school	72 (61.54)	0.14	27 (61.36)	0.62	17 (58.62)	0.59	12 (52.17)	0.52	48 (60.76)	0.55	114 (55.9)
From friends	6 (5.13)		1 (2.27)		0		2 (8.70)		3 (3.80)		10 (4.9)
Home cooked food	39 (33.33)		16 (36.36)		12 (41.38)		9 (39.13)		28 (35.44)		80 (39.2)
**Wash hands regularly with soap before eating**											
No	5 (4.27)	0.79	1 (2.27)	0.20	0	0.43	2 (8.70)	0.52	2 (2.53)	0.48	10 (4.9)
Yes	61 (52.14)		29 (65.91)		18 (62.07)		11 (47.83)		42 (53.16)		109 (53.4)
Sometimes	51 (43.59)		14 (31.82)		11 (37.93)		10 (43.48)		35 (44.30)		85 (41.7)
**Biting fingernails or sucking thumb**											
No	35 (29.91)	0.92	19 (43.18)	0.04	10 (34.48)	0.68	8 (34.78)	0.85	30 (37.97)	0.14	61 (29.9)
Yes	27 (23.08)		5 (11.36)		7 (24.14)		5 (21.74)		16 (20.25)		45 (22.1)
Sometimes	55 (47.01)		20 (45.45)		12 (41.38)		10 (43.48)		33 (41.77)		98 (48.0)
**Had diarrhea, stomach pain, vomited, or lost appetite in the past, what treatment was used**											
Herbal medicine	47 (40.17)	0.50	12 (27.27)	0.31	14 (48.28)	0.20	10 (43.48)	0.81	28 (35.44)	0.68	75 (36.8)
Orthodox medicine	64 (54.70)		30 (68.18)		15 (51.72)		12 (52.17)		48 (60.76)		118 (57.8)
None	6 (5.13)		2 (4.55)		0		1 (4.35)		3 (3.80)		11 (5.4)
**Symptoms experienced in the last 6 months**											
Diarrhea	37 (31.62)	0.41	11 (25.00)	0.48	12 (41.38)	0.17	8 (34.78)	0.34	24 (30.38)	0.38	59 (28.9)
Loss of appetite	17 (14.53)		10 (22.73)		3 (10.34)		4 (17.39)		9 (11.39)		30 (14.7)
Nausea/vomiting	13 (11.11)		5 (11.36)		2 (6.90)		3 (13.04)		16 (20.25)		30 (14.7)
Stomach pain	37 (31.62)		13 (29.55)		7 (24.14)		4 (17.39)		24 (30.38)		66 (32.4)
None	13 (11.11)		5 (11.36)		5 (17.24)		4 (17.39)		6 (7.59)		19 (9.3)
**Source of information on parasitic infection**											
Friends	15 (12.82)	0.68	6 (13.64)	0.57	5 (17.24)	0.87	1 (4.35)	0.69	12 (15.19)	0.83	25 (12.3)
Hospital worker	16 (13.68)		8 (18.18)		5 (17.24)		5 (21.74)		13 (16.49)		34 (16.7)
Media	26 (22.22)		12 (27.27)		7 (24.14)		4 (17.39)		15 (18.99)		45 (22.0)
Teachers	55 (47.01)		15 (34.09)		11 (37.93)		12 (52.17)		35 (44.30)		90 (44.1)
Others	5 (4.27)		3 (6.82)		1 (3.45)		1 (4.35)		4 (5.06)		10 (4.9)

Abbreviations: KVIP, Kumasi Ventilated Improved Pit; WC, water closet.

**FIGURE 2:**
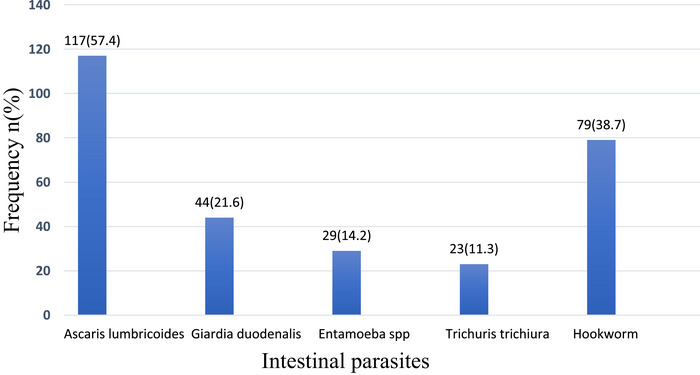
Prevalence of parasitic infections among the children.

**FIGURE 3:**
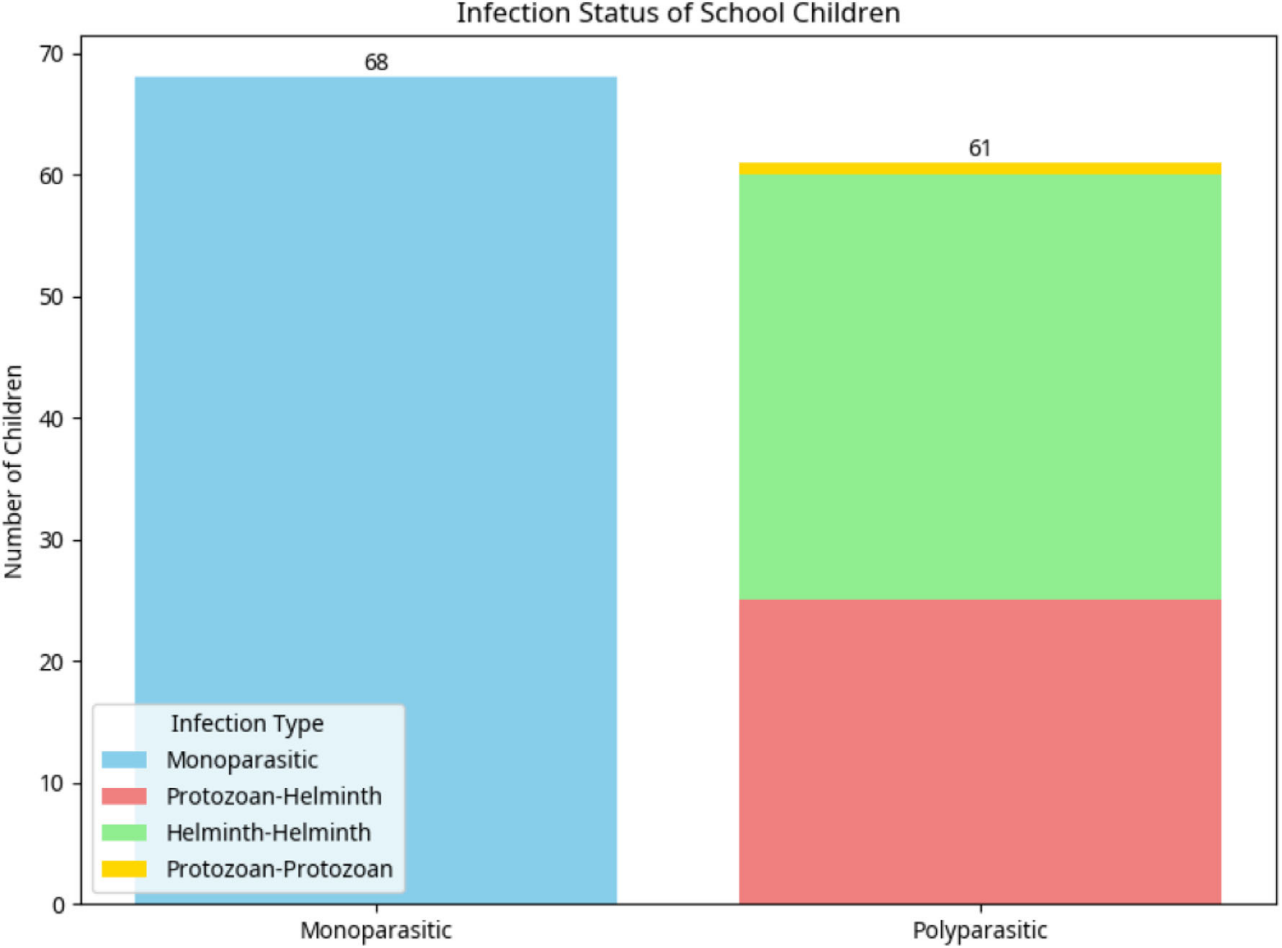
Intersection of parasitic infections.

**FIGURE 4:**
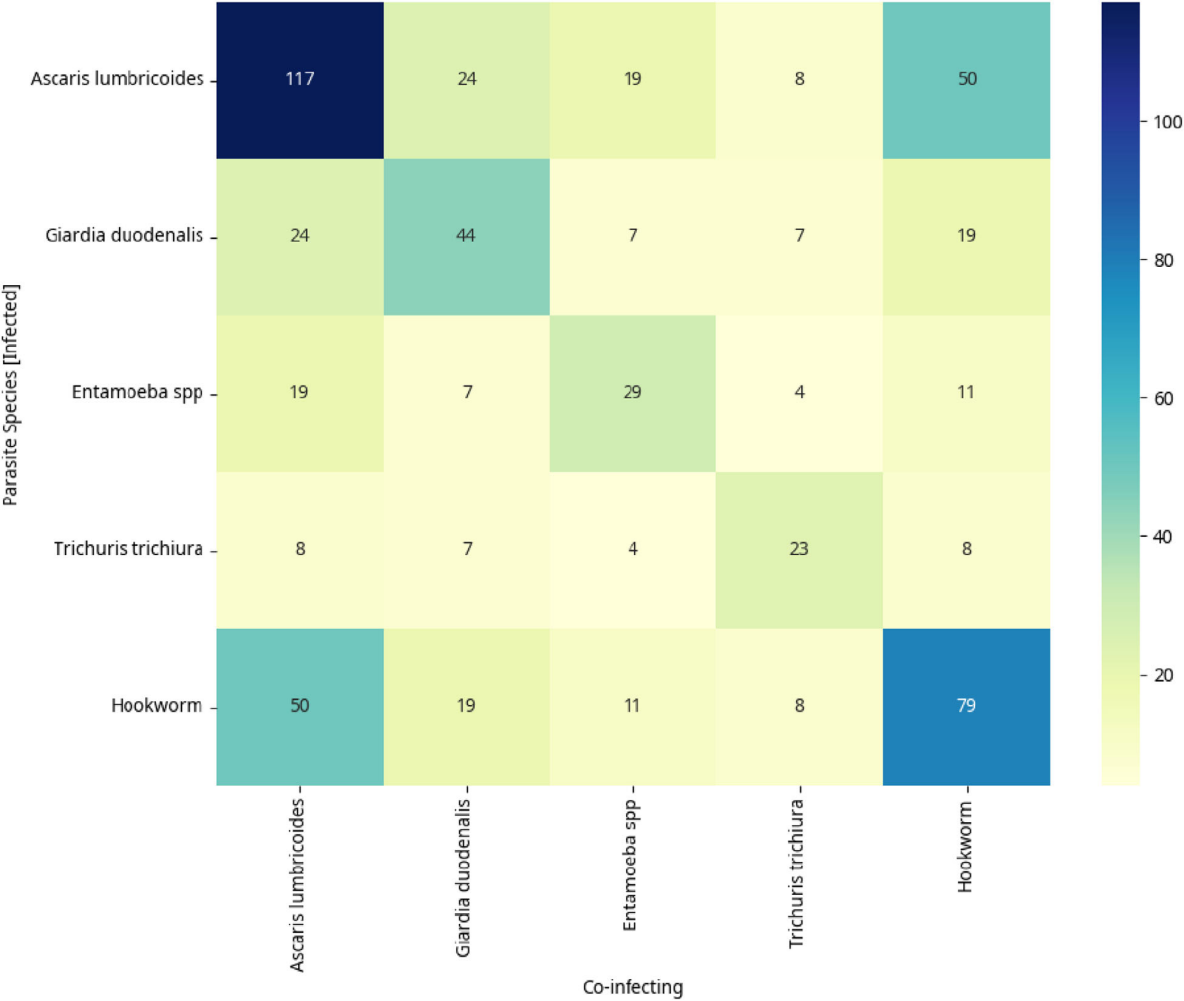
Heat map of coinfection counts among parasitic species.

Figure 2 shows the five different intestinal parasites that were commonly detected in the stool specimens of the children. The overall prevalence of monoparasitic infections among the study population was 33.33%. The prevalence of parasite species was *A. lumbricoides* (57.4%), *G. duodenalis* (21.6%), *Entamoeba* spp *(E. histolytica/dispar)* (14.2%), *T. trichiura* (11.3%), and Hookworm (38.7%). The intersection of parasitic infections and coinfections of the parasites in each individual are shown in Figures 3 and 4, respectively.

Univariate and multivariate analyses identified distinct sociodemographic, behavioral, and environmental risk factors associated with mono‐ and polyparasitic infections among children in the rural study area (Tables 2 and 3). *A. lumbricoides* infection risk was significantly increased with younger age (cOR = 1.11, *p* < 0.03; aOR = 1.13, 95% CI: 1.02–1.26, *p* = 0.019), likely reflecting naive hygiene practices in younger children. Home‐cooked meals exhibited a protective effect, reducing odds of infection in both (cOR = 0.55, 95% CI: 0.31–0.99, *p* < 0.05) and multivariate models (aOR = 0.49, 95% CI: 0.27–0.90, *p* = 0.022), suggesting reduced exposure to contaminated street food. For *G. duodenalis*, male gender was associated with elevated risk (cOR = 2.21, 95% CI: 1.02–4.79, *p* < 0.05; aOR = 2.20, *p* = 0.051), potentially due to gendered differences in hygiene or outdoor activities. The use of Kumasi Ventilated Improved Pit (KVIP) latrines (cOR = 10.19, 95% CI: 1.25–82.90, *p* < 0.03) and pit latrines at school (cOR = 11.11, 95% CI: 1.40–88.13, *p* < 0.02) paradoxically increased risk, possibly due to overcrowding, poor maintenance, or contamination. Fingernail‐biting or thumb‐sucking was linked to reduced odds (cOR = 0.28, *p* < 0.02), though this counterintuitive finding warrants further investigation for potential misclassification or confounding. Environmental exposures, including untreated river/stream water (cOR = 3.56, *p* < 0.02) and pipe‐borne water (cOR = 2.94, *p* < 0.05), heightened risk of IPI, emphasizing the role of unsafe water in transmission. *Entamoeba* spp. (*E. histolytica/dispar*) infection was linked to parental occupation (farming) and was protective (cOR = 0.18, 95% CI: 0.04–0.72, *p* < 0.02; aOR = 0.17, *p* = 0.022), potentially reflecting socioeconomic or hygiene practices unique to farming households. Awareness of intestinal parasites (cOR = 0.38, *p* < 0.02) and household toilet access (cOR = 0.35, *p* < 0.05) reduced risk, underscoring the importance of health education and sanitation infrastructure. Although KVIP latrine use at school reduced *T. trichiura* risk (cOR = 0.21, *p* < 0.03), suggesting a possible improvement in sanitation, fingernail‐biting or thumb‐sucking habits showed no significant association with hookworm infection (cOR = 0.52, *p* < 0.05), contrasting with typical fecal‐oral transmission pathways and suggesting context‐specific behavioral dynamics.

**TABLE 2:** The distributions of associated risk factors with the intestinal parasitic infections among school children.

	*Ascaris lumbricoides*			*Giardia duodenalis*			*Entamoeba histolytica/dispar*			*Trichuris trichuira*			Hookworm		
Characteristics	Odds ratio	95% CI	*p* value	Odds ratio	95% CI	*p* value	Odds ratio	95% CI	*p* value	Odds ratio	95% CI	*p* value	Odds ratio	95% CI	*p* value
**Gender**															
Female	Ref.														
Male	0.76	0.42–1.36	0.36	2.21	1.02–4.79	**0.05**	0.76	0.34–1.69	0.50	0.69	0.29–1.68	0.42	1.79	0.97–3.28	0.06
**Parents educational level**															
No formal education	Ref.														
Basic education	1.64	0.51–5.29	0.40	0.73	0.20–2.60	0.63	1.43	0.17–12.24	0.75	0.97	0.11–8.59	0.98	0.57	0.18–1.86	0.36
Secondary education	1.53	0.47–4.92	0.48	0.52	0.14–1.91	0.33	2.79	0.34–23.10	0.34	2.03	0.24–17.02	0.52	0.93	0.29–3.00	0.91
Tertiary education	2.90	0.41–21.0	0.29	—	—	—	2.00	0.10–38.10	0.65	4.8	0.35–66.18	0.24	0.47	0.06–3.36	0.45
**Parents occupation**															
Unemployed	Ref.														
Civil servant	1.38	0.31–6.16	0.67	0.29	0.05–1.50	0.14	0.20	0.04–1.18	0.08	0.35	0.04–2.91	0.33	1.63	0.36–7.23	0.52
Farmer	2.06	0.55–7.74	0.28	0.38	0.10–1.46	0.16	0.18	0.04–0.72	**0.02**	0.56	0.11–2.92	0.49	0.88	0.23–3.29	0.84
Trader	2.63	0.66–10.46	0.17	0.46	0.11–1.91	0.29	0.33	0.08–1.41	0.14	0.40	0.06–2.43	0.32	0.86	0.22–3.42	0.83
**Awareness of intestinal parasitic infection**															
No	Ref.														
Yes	0.81	0.43–1.53	0.52	1.29	0.59–2.84	0.53	0.38	0.17–0.85	**0.02**	0.42	0.17–1.02	0.06	0.80	0.43–1.51	0.50
**Often become unwell**															
Every 4 months	Ref.														
Every 8 months	1.12	0.46–2.75	0.80	1.20	0.40–3.57	0.82	2.58	0.56–11.98	0.23	0.38	0.11–1.25	0.11	0.86	0.35–2.13	0.74
Every 12 months	1.21	0.48–3.03	0.69	0.88	0.28–2.73	0.74	1.22	0.24–6.21	0.81	0.48	0.14–1.61	0.24	0.89	0.35–2.25	0.80
**Type of toilet facility at home**															
Open defecation	Ref.														
Public toilet	0.50	0.19–1.34	0.17	0.68	0.20–2.35	0.54	0.38	0.11–1.22	0.10	1.07	0.25–4.55	0.93	2.11	0.73–6.14	0.17
Within house	0.86	0.35–2.10	0.74	1.33	0.46–3.86	0.60	0.35	0.13–0.99	**0.05**	0.86	0.23–3.28	0.82	2.21	0.82–5.94	0.12
**Type of toilet facility at school**															
Open defecation	Ref.														
KVIP	2.33	0.91–5.97	0.08	10.19	1.25–82.90	**0.03**	0.24	0.06–0.88	**0.03**	0.21	0.05–0.89	**0.03**	1.47	0.58–3.73	0.42
Pit latrine	1.32	0.55–3.15	0.53	11.11	1.40–88.13	**0.02**	0.28	0.09–0.90	**0.03**	0.33	0.10–1.10	0.07	1.01	0.41–2.49	0.98
WC	1.03	0.43–2.50	0.94	7.40	0.91–60.28	0.06	0.63	0.22–1.79	0.39	0.44	0.14–1.41	0.17	0.95	0.38–2.39	0.92
**Main source of drinking water at home**															
Borehole/well	Ref.														
Pipe‐borne water	0.75	0.36–1.58	0.45	2.94	1.00–8.64	**0.05**	0.45	0.16–1.29	0.14	1.77	0.43–7.23	0.43	0.63	0.30–1.32	0.22
River/stream	0.75	0.35–1.61	0.46	3.56	1.20–10.52	**0.02**	0.62	0.22–1.71	0.35	3.13	0.81–12.13	0.10	0.73	0.34–1.57	0.42
Sachet water	0.92	0.34–2.46	0.87	2.25	0.58–8.68	0.24	0.76	0.21–2.73	0.68	2.14	0.40–11.49	0.38	0.66	0.25–1.78	0.41
**Source of food while at school**															
Buy from the street/school	Ref.														
From friends	0.87	0.23–3.29	0.84	0.36	0.04–2.97	0.34	—	—	—	2.13	0.40–11.23	0.38	0.59	0.14–2.40	0.46
Home cooked food	0.55	0.31–0.99	**0.05**	0.81	0.40–1.62	0.54	1.01	0.45–2.25	0.99	1.08	0.43–2.70	0.87	0.74	0.41–1.34	0.32
**Wash hands regularly with soap before eating**															
No	Ref.														
Yes	1.50	0.40–5.60	0.55	3.26	0.39–27.03	0.27	—	—	—	0.45	0.08–2.40	0.35	2.80	0.56–14.04	0.21
Sometimes	1.30	0.35–4.67	0.72	1.77	0.21–15.22	0.60	0.75	0.33–1.69	0.49	0.53	0.10–2.89	0.47	2.51	0.51–12.43	0.26
**Biting fingernails or sucking thumb**															
No	Ref.														
Yes	0.95	0.50–1.81	0.88	0.28	0.09–0.81	0.02	0.93	0.32–2.70	0.91	0.83	0.25–2.73	0.76	0.57	0.26–1.26	0.16
Sometimes	1.11	0.51–2.44	0.79	0.57	0.27–1.18	0.13	0.71	0.29–1.77	0.46	0.75	0.28–2.03	0.58	0.52	0.27–1.01	**0.05**
**Had diarrhea, stomach pain, vomited, or lost appetite in the past, what treatment was used**															
Herbal medicine	1.40	0.39–5.03	0.61	0.86	0.16–4.48	0.86	1.58	0.71–3.49	0.26	1.54	0.18–13.42	0.70	1.59	0.39–6.51	0.52
Orthodox medicine	0.99	0.28–3.43	0.98	1.53	0.31–7.53	0.60	—	—	—	1.13	0.13–9.68	0.91	1.83	0.46–7.27	0.39
None	Ref.														
**Symptoms experienced in the last 6 months**															
Diarrhea	0.78	0.26–2.34	0.65	0.64	0.19–2.17	0.48	0.71	0.21–2.39	0.59	0.59	0.15–2.23	0.44	1.49	0.49–4.47	0.48
Loss of appetite	0.60	0.18–2.02	0.41	0.56	0.14–2.28	0.42	0.31	0.06–1.50	0.15	0.58	0.13–2.66	0.48	0.93	0.27–3.23	0.91
Nausea/vomiting	0.35	0.11–1.18	0.09	1.4	0.39–5.01	0.61	0.2	0.03–1.17	0.07	0.42	0.08–2.12	0.29	2.48	0.74–8.28	0.14
Stomach pain	0.59	0.20–1.74	0.20	0.69	0.21–2.26	0.54	0.33	0.09–1.21	0.09	0.24	0.05–1.08	0.06	1.24	0.42–3.69	0.71
None	Ref.														
**Source of information on parasitic infection**															
Friends	1.5	0.34–6.58	0.59	0.74	0.14–3.79	0.72	2.25	0.23–22.27	0.49	0.38	0.02–6.70	0.51	1.38	0.31–6.16	0.67
Hospital worker	0.89	0.22–3.66	0.87	0.72	0.15–3.46	0.68	1.55	0.16–15.16	0.71	1.55	0.16–15.16	0.71	0.93	0.22–3.94	0.92
Media	1.37	0.35–5.42	0.66	0.85	0.19–3.84	0.83	1.66	0.18–15.31	0.66	0.88	0.09–8.87	0.91	0.75	0.18–3.08	0.69
Teachers	1.57	0.42–5.84	0.50	0.47	0.11–2.02	0.31	1.25	0.14–10.93	0.84	1.38	0.16–11.99	0.77	0.95	0.25–3.64	0.95
Others	Ref.														

*Note:* Statistically significant: *p* value ≤0.05.

Abbreviations: KVIP, Kumasi Ventilated Improved Pit; WC, water closet.

**TABLE 3:** The prevalence of Intestinal parasitic infections among school children.

Parasitic infection	Prevalence	Confidence interval
*Ascaris lumbricoides*	0.57	0.50–0.64
*Giardia duodenalis*	0.22	0.16–0.27
*Entamoeba dispar/histolytica*	0.14	0.10–0.20
*Trichuris trichuira*	0.11	0.07–0.16
Hookworm	0.39	0.32–0.46

Table 4 presents the adjusted odds ratios (AOR) and 95% CIs for the associations between specific intestinal parasite infections and various contextual factors among the school children. There were significant associations between *A. lumbricoides* infection and the source of food at school and the type of toilet used. Children who consumed home‐cooked food at school had significantly lower odds of *A. lumbricoides* infection compared to those who bought food from the street/school (AOR = 0.49, 95% CI: 0.27–0.90, *p* = 0.02). The use of water closet (WC) facilities was associated with significantly reduced odds of *A. lumbricoides* infection compared to KVIP latrines (AOR = 0.40, 95% CI: 0.18–0.91, *p* = 0.03). Although open defecation (AOR = 0.43, 95% CI: 0.17–1.09, *p* = 0.08) and pit latrine use (AOR = 0.54, 95% CI: 0.25–1.19, *p* = 0.13) also showed lower odds, these associations were not statistically significant.

**TABLE 4:** The associations of intestinal parasite infections with the assessed contextual associated factors.

*Ascaris lumbricoides*	Adjusted odds ratio	95% CI	*p* value
**Source of food at school**			
From friends	0.74	0.20–2.70	0.65
Home cooked food	0.49	0.27–0.90	0.02**
Buy from street/school	Ref.		
**Type of toilet**			
Open defecation	0.43	0.17–1.09	0.08
Pit latrine	0.54	0.25–1.19	0.13
WC	0.40	0.18–0.91	0.03**
KVIP	Ref.		

***Giardia duodenalis***	**Adjusted odds ratio**	**95% CI**	***p* value**
**Gender**			
Male	2.20	0.99–4.85	0.05**
Female	Ref.		
**Biting fingernails bite**			
Sometimes	0.55	0.26–1.14	0.11
Yes	0.28	0.09–0.84	0.02**
No	Ref.		

***Entamoeba dispar/histolytica***	**Odds ratio**	**95% CI**	***p* value**
**Awareness of intestinal parasitic infection**			
Yes	0.37	0.15–0.86	0.02**
No	Ref.		
**Parents occupation**			
Civil servant	0.23	0.04–1.39	0.11
Farmer	0.17	0.04–0.78	0.02**
Trader	0.33	0.07–1.55	0.16
Unemployed	Ref.		

*Note:* Statistically significant **p value ≤0.05.

Abbreviations: KVIP, Kumasi Ventilated Improved Pit; WC, water closet.

Furthermore, a significant association between *G. duodenalis* infection, gender, and fingernail biting habits was observed. Male children had a higher likelihood of *G. duodenalis* infection compared to female children (AOR = 2.20, 95% CI: 0.99–4.85, *p* = 0.05). Children who reported biting their fingernails “Yes” had significantly lower odds of *G. duodenalis* infection compared to those who did not bite their fingernails (AOR = 0.28, 95% CI: 0.09–0.84, *p* = 0.02). The “Sometimes” category for fingernail biting did not show a statistically significant association (AOR = 0.55, 95% CI: 0.26–1.14, *p* = 0.11).

For *E. dispar/histolytica* infection, significant associations were identified with awareness of IPI and parents' occupation. Children who were aware of IPIs had significantly lower odds of *E. dispar/histolytica* infection (AOR = 0.37, 95% CI: 0.15–0.86, *p* = 0.02). Among parents' occupations, having a parent who was a farmer was associated with significantly lower odds of *E. dispar/histolytica* infection compared to unemployed parents (AOR = 0.17, 95% CI: 0.04–0.78, *p* = 0.02). Other parental occupations such as civil servant (AOR = 0.23, 95% CI: 0.04–1.39, *p* = 0.11) and trader (AOR = 0.33, 95% CI: 0.07–1.55, *p* = 0.16) did not show statistically significant associations.

Of 204 school children, 129 (63.23%) had mono or polyparasitic infections. The prevalence of monoparasitic infection was 33.3% (*n* = 68). Detected intestinal parasites included: *A. lumbricoides* (57.4%, *n* = 117), Hookworm (38.7%, *n* = 79), *G. duodenalis* (21.6%, *n* = 44), *E. dispar/histolytica* (14.2%, *n* = 29), and *T. trichiura* (11.3%, *n* = 23). Polyparasitism was observed with double, triple, and quadruple infections detected in the children. Twenty‐five (19.38%) protozoan–helminth infections, 35 (27.13%) helminth–helminth infections, and 1 (0.78%) protozoan–protozoan infection were detected. Most infections, 30 (23.26%), involved *A. lumbricoides* and Hookworm, followed by *G. duodenalis* and *A. lumbricoides* (9, 6.98%), 7 (5.43%) *E. dispar/histolytica* and *A. lumbricoides*, and the least was *E. dispar/histolytica* and *T. trichiura* (1, 0.78%).

Triple infections included: *G. duodenalis* + Hookworm + *A. lumbricoides* (9, 6.98%), *E. dispar*/*histolytica* + Hookworm + *A. lumbricoides* (5, 3.88%), *T. trichiura* + *G. duodenalis* + Hookworm (3, 2.33%), *E. dispar/histolytica* + *G. duodenalis* + *A. lumbricoides* (2, 1.55%), and *T. trichiura* + Hookworm + *A. lumbricoides* (2, 1.55%). Additionally, one (0.78%) child had the following combinations: *T. trichiura* + *G. duodenalis* + *A. lumbricoides*, *T. trichiura* + *E. dispar/histolytica* + *A. lumbricoides*, and *T. trichiura* + *E. dispar/histolytica* + *G. duodenalis*.

Quadruple intestinal infections involved *Entamoeba* spp. + *G. duodenalis* + Hookworm + *A. lumbricoides* in 3 (2.33%) children and *T. trichiura* + *E. dispar/histolytica* + Hookworm + *A. lumbricoides* in 1 (0.78%) child (Figure 3).

## 4 Discussion

This study revealed a high burden (63.2%) of IPIs among schoolchildren at Tokuroano in the Krachi East Municipality of the Oti Region of Ghana, detected using microscopy. Although microscopy is the primary diagnostic method in resource‐limited settings like Ghana, its sensitivity for low‐intensity infections or morphologically similar species is lower compared to PCR‐based assays. The observed IPI prevalence is significantly higher than reported in previous studies from Accra [15, 16], Ho Municipality [19], and the neighboring Kadjebi District of the Oti Region [20]. This high prevalence aligns with findings from other hyperendemic transmission zones in sub‐Saharan Africa, including Burkina Faso [35], Togo [36], Nigeria [9, 10], and Kenya [37, 38]. Several contextual factors likely contribute to this elevated prevalence, such as widespread open defecation, unsafe water sources, poor hygiene practices, and environmental contamination, all of which increase exposure to both helminths and protozoa [19, 27]. Furthermore, reliance on microscopy and single‐stool sampling may underestimate true prevalence, suggesting that actual transmission intensity may be even higher.

The prevalence of *A. lumbricoides* (57.4%) and Hookworm (38.7%), in this study, is inconsistent with findings from other regions of Ghana, such as the Volta [19, 39] and Greater Accra Regions [15, 16]. The findings are also consistent with similar studies conducted in the Kpandai district [40], Kintampo North Municipality, and Kintampo South district, located in the Middle belt of Ghana [41]. Studies in Kenya [37] and Ethiopia [42, 43] also had similar findings. Polyparasitism, including dual and triple infections involving these species, was common, underscoring overlapping exposure routes, particularly for STHs in contaminated environments [43]. This pattern is characteristic of areas with poor sanitation, inadequate access to clean water, and limited hygiene practices, which facilitate the transmission of various intestinal parasites, as observed in Nepal [44], Ethiopia [45, 46], Cote d'Ivoire [47], Sudan [48], and Bolivia [2]. *A. lumbricoides* and hookworm were the predominant parasites detected with common transmission routes [49, 50]. The lack of significant clustering among *A. lumbricoides* and hookworm suggests that exposure risks, such as barefoot contact with contaminated soil or consumption of unwashed produce, are widespread rather than specific to individual parasites. The protective effect of home‐cooked meals against *A. lumbricoides* highlights the risks associated with street food, which can be contaminated due to inadequate hygiene during preparation or vending. This observation is consistent with studies linking street food to diarrheal diseases in similar settings.

*G. duodenalis* (21.6%) and *E. histolytica/dispar* (14.2%) represent protozoal infections linked to unsafe drinking water and poor food hygiene. The prevalence rates for these protozoa in this study are inconsistent with those reported in Chorkor, Greater Accra Region [15], and Ho Municipality [19]and are higher than those found in some studies in Sudan [51] and Togo [36]. Contrary to typical global patterns, male children exhibited a significantly higher risk of *Giardia* infection, possibly due to behavioral differences like increased outdoor exposure or less frequent handwashing. The vulnerability of younger children to *A. lumbricoides* emphasizes the importance of early hygiene education. The co‐detection of protozoa (*G. duodenalis*, *E. dispar/histolytica*) alongside helminths points to fecal‐oral transmission pathways exacerbated by inadequate waste management and contaminated water sources [50, 52].These findings support existing studies in similar low‐resource settings, where polyparasitism arises from systemic infrastructural deficiencies rather than direct biological interactions between parasites.

The high prevalence of polyparasitism suggests the inadequacy of single‐pathogen control strategies, inadequate sanitation, and safe water. Integrated interventions, including MDA, improved WASH infrastructure, health education, and community engagement are necessary to reduce environmental transmission of the burden of fecal‐oral diseases [53]. Although MDA effectively controls STHs, expanding it to include antiprotozoal drugs is critical for comprehensive parasitic control and transmission disruption, especially in endemic areas [54].

An unexpected finding was the significantly reduced odds of *Entamoeba* infection among children of farming parents. This contradicts typical associations of farming with increased zoonotic or soil‐based transmission. A possible explanation is improved hygiene practices within farming households, potentially due to habitual outdoor work, more regulated mealtimes, or greater access to home‐prepared meals, which this study also identified as protective against *Ascaris* infection. Farming families may also be more likely to consume home‐cooked meals, thereby reducing exposure to contaminated street food, a known risk factor for faeco‐oral parasites.

Sanitation infrastructure played a nuanced role. Although the use of improved toilets like WCs was protective against *Ascaris* infection, KVIP and pit latrines at school were paradoxically associated with an increased risk of *Giardia* infection. This contrasts with global evidence linking improved sanitation to reduced transmission. This discrepancy might be attributed to overcrowding or poor maintenance of these facilities, fostering contamination, or misclassification bias, where “improved” infrastructure does not equate to safe usage practices, or residual confounding from unmeasured variables such as water quality or handwashing compliance.

Surprisingly, fingernail‐biting or thumb‐sucking showed a protective association against *Giardia* infection, which contradicts its usual role as a risk factor in feco‐oral transmission. This counterintuitive finding may reflect reporting bias, misclassification, or unmeasured confounding, where children prohibited from nail‐biting might engage in other high‐risk behaviors.

The high burden of polyparasitism, including dual and triple infections involving helminths and protozoa, suggests that interventions focusing on a single pathogen are insufficient [43]. Although MDA campaigns with albendazole or mebendazole target helminths, they do not address protozoan infections. In Ghana, MDA programs under the Neglected Tropical Diseases Program (NTDP) primarily focus on school‐based deworming but often lack consistent integration of WASH improvements or treatment for protozoa like *Giardia* and *E. dispar/histolytica*.

This study's findings advocate for a holistic, integrated control strategy that combines MDA with sustainable WASH interventions and behavioral change communication. Frameworks, such as the Ghana National Sanitation Policy and the School Health Education Programme (SHEP), can guide these actions. Integrating parasite‐specific education into school curricula and fostering community‐led sanitation efforts are essential to break transmission cycles.

Furthermore, the “One Health” approach, endorsed by the WHO, is highly relevant given the probable zoonotic component in transmission. Interventions addressing environmental contamination from animal reservoirs, alongside human behavioral and infrastructure changes, are crucial. Without concurrent improvements in sanitation, water access, and hygiene behavior, reinfection after deworming is inevitable. Studies demonstrate that One Health interventions effectively reduce the prevalence of intestinal parasites and disease risks in at‐risk populations by promoting community empowerment, fostering multi‐sectoral collaboration, and enhancing sanitation practices [55, 56]. Addressing challenges related to socioeconomic determinants, such as household crowding and poverty, is equally necessary to reduce exposure. Without tackling these systemic challenges, IPIs are likely to remain a persistent threat to child health, particularly in rural Ghana.

## 5 Conclusion

This study highlights mono and polyparasitism as a major public health challenge in the area. *A. lumbricoides*, Hookworm, and *G. duodenalis* were the primary high‐burden parasites in single and co‐infections. The lack of significant paired associations suggests diverse transmission pathways or overlapping risk factors. These findings emphasize the urgent need for integrated deworming programs, improved sanitation, and targeted health education to reduce morbidity associated with parasitic infections in vulnerable pediatric populations.

## 6 Limitations of the Study

A single‐stool sample collected within 24 h may have reduced detection rates of some intestinal parasites. Additionally, sampling between 8:30 a.m. and 9:30 a.m. may be suboptimal for detecting certain species due to their diurnal and intermittent shedding patterns. The study was conducted in a single rural community using a cross‐sectional study design. This hindered causal inferences and generalizability of the study outcome. The use of microscopy for parasite detection in single‐fresh‐stool samples risks underestimating true infection prevalence due to its low sensitivity compared to molecular techniques and potential missed detections from intermittent pathogen shedding or low‐burden infections (Supporting Table).

## Author Contributions

Christopher Yaw Dumevi conceptualized, designed the study, wrote the first draft of the manuscript, reviewed, and edited the manuscript. Prince Wise Amekudi did the data collection. Nana Aba Setorwu Eyeson and Ezekiel Kofi Vicar analyzed the data. Hugette Naa Ayele Aryee, Joyce Junior Asiamah, James‐Paul Kretchy, Simon Sovoe, Saviour Kweku Adjenti, Nicholas T. K. D. Dayie, George Boateng Kyei, Patience B. Tetteh‐Quarcoo, Irene Ayi, Patrick F. Ayeh‐Kumi reviewed and edited the manuscript.

## Funding

The authors have nothing to report.

## Conflicts of Interest

The authors declare no conflicts of interest.

## Ethics Statement

Ethical approval of this study was granted by the Institutional Review Board of Central University, Miotso, Accra (Protocol ID: CUIRB/05/06/24). Permission was sought and approval granted by the head of the school. Written informed consent was provided by the parents and legal guardians of the pupils, and the children assented after explaining the study objectives, risks, benefits, right to refuse. and confidentiality. Participation was purely based on volunteerism. Personal data of the study participants were treated with strict confidentiality and anonymity.

## Transparency Statement

I, Christopher Yaw Dumevi, the Lead author, affirms that this manuscript is an honest, accurate, and transparent account of the study being reported; that no important aspects of the study have been omitted; and that any discrepancies from the study as planned (and, if relevant, registered) have been explained. All authors have read and approved the final version of the manuscript, and the Corresponding author has full access to all of the data in this study and takes complete responsibility for the integrity of the data and the accuracy of the data analysis.

## Supporting information




**Table S2:** Crude odds ratios, confidence intervals, and *p* values
Table 3a
Table 3b

## Data Availability

The authors confirm that all data supporting the findings of the study are available in the article. Any other information will be made available by requesting through the corresponding author.
