# Supplementary material for: Prevalence of Intestinal Parasitic Infections and Associated Factors Among Rural Ghanaian School Children: A Cross‐Sectional Study in Tokuroano, Krachi East Municipality
Source: Public Health Chall. 2026 Jan 29;5(1):e70176. doi: 10.1002/puh2.70176 (PMC12854096; doi:10.1002/puh2.70176)
Supplement: Supplementary file 1 — Table S2: Crude odds ratios, confidence intervals, and p values Table 3a Table 3b [file PUH2-5-e70176-s001.docx]

**Table 2: Crude Odds ratios, Confidence Intervals and P-values**

| **Characteristics** |  |  |  |  |  |  |  |  |  |  |  |  |  |  |  |  |
| --- | --- | --- | --- | --- | --- | --- | --- | --- | --- | --- | --- | --- | --- | --- | --- | --- |
|  | ***Ascaris lumbricoides*** | | | ***Giardia duodenale*** | | | ***Entamoeba histolytica/dispar*** | | | ***Trichuris trichuira*** | | | **Hookworm** | | | |
|  | Odds ratio | 95% CI | p-value | Odds ratio | 95% CI | p-value | Odds ratio | 95% CI | p-value | Odds ratio | 95% CI | p-value | Odds ratio | 95% CI | p-value |  |
| **Gender** |  |  |  |  |  |  |  |  |  |  |  |  |  |  |  |  |
| Female | Ref. |  |  |  |  |  |  |  |  |  |  |  |  |  |  |  |
| Male | 0.76 | 0.42 - 1.36 | 0.36 | 2.21 | 1.02 - 4.79 | **0.05** | 0.76 | 0.34 - 1.69 | 0.50 | 0.69 | 0.29 - 1.68 | 0.42 | 1.79 | 0.97 - 3.28 | 0.06 |  |
| **Parents Educational Level** |  |  |  |  |  |  |  |  |  |  |  |  |  |  |  |  |
| No Formal Education | Ref. |  |  |  |  |  |  |  |  |  |  |  |  |  |  |  |
| Basic Education | 1.64 | 0.51 - 5.29 | 0.40 | 0.73 | 0.20 -2.60 | 0.63 | 1.43 | 0.17 - 12.24 | 0.75 | 0.97 | 0.11 - 8.59 | 0.98 | 0.57 | 0.18 - 1.86 | 0.36 |  |
| Secondary Education | 1.53 | 0.47 - 4.92 | 0.48 | 0.52 | 0.14 - 1.91 | 0.33 | 2.79 | 0.34 - 23.10 | 0.34 | 2.03 | 0.24 - 17.02 | 0.52 | 0.93 | 0.29 - 3.00 | 0.91 |  |
| Tertiary Education | 2.90 | 0.41 - 21.0 | 0.29 | - | - | - | 2.00 | 0.10 - 38.10 | 0.65 | 4.8 | 0.35 - 66.18 | 0.24 | 0.47 | 0.06 - 3.36 | 0.45 |  |
| **Parents Occupation** |  |  |  |  |  |  |  |  |  |  |  |  |  |  |  |  |
| Unemployed | Ref. |  |  |  |  |  |  |  |  |  |  |  |  |  |  |  |
| Civil Servant | 1.38 | 0.31 - 6.16 | 0.67 | 0.29 | 0.05 - 1.50 | 0.14 | 0.20 | 0.04 - 1.18 | 0.08 | 0.35 | 0.04 - 2.91 | 0.33 | 1.63 | 0.36 - 7.23 | 0.52 |  |
| Farmer | 2.06 | 0.55 - 7.74 | 0.28 | 0.38 | 0.10 - 1.46 | 0.16 | 0.18 | 0.04 - 0.72 | **0.02** | 0.56 | 0.11 - 2.92 | 0.49 | 0.88 | 0.23 - 3.29 | 0.84 |  |
| Trader | 2.63 | 0.66 - 10.46 | 0.17 | 0.46 | 0.11 - 1.91 | 0.29 | 0.33 | 0.08 - 1.41 | 0.14 | 0.40 | 0.06 - 2.43 | 0.32 | 0.86 | 0.22 - 3.42 | 0.83 |  |
| **Awareness of Intestinal Parasitic Infection** |  |  |  |  |  |  |  |  |  |  |  |  |  |  |  |  |
| No | Ref. |  |  |  |  |  |  |  |  |  |  |  |  |  |  |  |
| Yes | 0.81 | 0.43 - 1.53 | 0.52 | 1.29 | 0.59 -2.84 | 0.53 | 0.38 | 0.17 - 0.85 | **0.02** | 0.42 | 0.17 - 1.02 | 0.06 | 0.80 | 0.43 - 1.51 | 0.50 |  |
| **Often become unwell** |  |  |  |  |  |  |  |  |  |  |  |  |  |  |  |  |
| Every 4 months | Ref. |  |  |  |  |  |  |  |  |  |  |  |  |  |  |  |
| Every 8 months | 1.12 | 0.46 - 2.75 | 0.80 | 1.20 | 0.40 - 3.57 | 0.82 | 2.58 | 0.56 - 11.98 | 0.23 | 0.38 | 0.11 - 1.25 | 0.11 | 0.86 | 0.35 - 2.13 | 0.74 |  |
| Every 12 months | 1.21 | 0.48 - 3.03 | 0.69 | 0.88 | 0.28 - 2.73 | 0.74 | 1.22 | 0.24 - 6.21 | 0.81 | 0.48 | 0.14 - 1.61 | 0.24 | 0.89 | 0.35 - 2.25 | 0.80 |  |
| **Type of Toilet Facility at Home** |  |  |  |  |  |  |  |  |  |  |  |  |  |  |  |  |
| Open defaecation | Ref. |  |  |  |  |  |  |  |  |  |  |  |  |  |  |  |
| Public Toilet | 0.50 | 0.19 - 1.34 | 0.17 | 0.68 | 0.20 - 2.35 | 0.54 | 0.38 | 0.11 - 1.22 | 0.10 | 1.07 | 0.25 - 4.55 | 0.93 | 2.11 | 0.73 - 6.14 | 0.17 |  |
| Within House | 0.86 | 0.35 - 2.10 | 0.74 | 1.33 | 0.46 - 3.86 | 0.60 | 0.35 | 0.13 - 0.99 | **0.05** | 0.86 | 0.23 - 3.28 | 0.82 | 2.21 | 0.82 - 5.94 | 0.12 |  |
| **Type of Toilet Facility at School** |  |  |  |  |  |  |  |  |  |  |  |  |  |  |  |  |
| Open defaecation | Ref. |  |  |  |  |  |  |  |  |  |  |  |  |  |  |  |
| KVIP | 2.33 | 0.91 - 5.97 | 0.08 | 10.19 | 1.25 - 82.90 | **0.03** | 0.24 | 0.06 - 0.88 | **0.03** | 0.21 | 0.05 - 0.89 | **0.03** | 1.47 | 0.58 - 3.73 | 0.42 |  |
| Pit Latrine | 1.32 | 0.55 - 3.15 | 0.53 | 11.11 | 1.40 - 88.13 | **0.02** | 0.28 | 0.09 - 0.90 | **0.03** | 0.33 | 0.10 - 1.10 | 0.07 | 1.01 | 0.41 - 2.49 | 0.98 |  |
| WC | 1.03 | 0.43 - 2.50 | 0.94 | 7.40 | 0.91 - 60.28 | 0.06 | 0.63 | 0.22 - 1.79 | 0.39 | 0.44 | 0.14 - 1.41 | 0.17 | 0.95 | 0.38 - 2.39 | 0.92 |  |
| **Main source of drinking water at Home** | | |  | |  |  | |  |  | |  |  | |  |  |  |
| Borehole/ Well | Ref. |  |  |  |  |  |  |  |  |  |  |  |  |  |  |  |
| Pipe-borne water | 0.75 | 0.36 - 1.58 | 0.45 | 2.94 | 1.00 - 8.64 | **0.05** | 0.45 | 0.16 - 1.29 | 0.14 | 1.77 | 0.43 - 7.23 | 0.43 | 0.63 | 0.30 - 1.32 | 0.22 |  |
| River/stream | 0.75 | 0.35 - 1.61 | 0.46 | 3.56 | 1.20 - 10.52 | **0.02** | 0.62 | 0.22 - 1.71 | 0.35 | 3.13 | 0.81 - 12.13 | 0.10 | 0.73 | 0.34 - 1.57 | 0.42 |  |
| Sachet water | 0.92 | 0.34 - 2.46 | 0.87 | 2.25 | 0.58 - 8.68 | 0.24 | 0.76 | 0.21 - 2.73 | 0.68 | 2.14 | 0.40 - 11.49 | 0.38 | 0.66 | 0.25 - 1.78 | 0.41 |  |
| **Source of Food while at School** |  |  |  |  |  |  |  |  |  |  |  |  |  |  |  |  |
| Buy from the street/School | Ref. |  |  |  |  |  |  |  |  |  |  |  |  |  |  |  |
| From friends | 0.87 | 0.23 - 3.29 | 0.84 | 0.36 | 0.04 - 2.97 | 0.34 | - | - | - | 2.13 | 0.40 - 11.23 | 0.38 | 0.59 | 0.14 - 2.40 | 0.46 |  |
| Home cooked food | 0.55 | 0.31 - 0.99 | **0.05** | 0.81 | 0.40 - 1.62 | 0.54 | 1.01 | 0.45 - 2.25 | 0.99 | 1.08 | 0.43 - 2.70 | 0.87 | 0.74 | 0.41 - 1.34 | 0.32 |  |
| **Wash hands regularly with soap before eating** |  |  |  |  |  |  |  |  |  |  |  |  |  |  |  |  |
| No | Ref. |  |  |  |  |  |  |  |  |  |  |  |  |  |  |  |
| Yes | 1.50 | 0.40 - 5.60 | 0.55 | 3.26 | 0.39 - 27.03 | 0.27 | - | - | - | 0.45 | 0.08 - 2.40 | 0.35 | 2.80 | 0.56 - 14.04 | 0.21 |  |
| Sometimes | 1.30 | 0.35 - 4.67 | 0.72 | 1.77 | 0.21 - 15.22 | 0.60 | 0.75 | 0.33 - 1.69 | 0.49 | 0.53 | 0.10 - 2.89 | 0.47 | 2.51 | 0.51 - 12.43 | 0.26 |  |
| **Biting Fingernails or sucking thumb** |  |  |  |  |  |  |  |  |  |  |  |  |  |  |  |  |
| No | Ref. |  |  |  |  |  |  |  |  |  |  |  |  |  |  |  |
| Yes | 0.95 | 0.50 - 1.81 | 0.88 | 0.28 | 0.09 - 0.81 | 0.02 | 0.93 | 0.32 - 2.70 | 0.91 | 0.83 | 0.25 - 2.73 | 0.76 | 0.57 | 0.26 - 1.26 | 0.16 |  |
| Sometimes | 1.11 | 0.51 - 2.44 | 0.79 | 0.57 | 0.27 - 1.18 | 0.13 | 0.71 | 0.29 - 1.77 | 0.46 | 0.75 | 0.28 - 2.03 | 0.58 | 0.52 | 0.27 - 1.01 | **0.05** |  |
| **Had Diarrhea, stomach pain, vomitted, or lost appetite in the past, what treatment was used** |  |  |  |  |  |  |  |  |  |  |  |  |  |  |  |  |
| Herbal Medicine | 1.40 | 0.39 - 5.03 | 0.61 | 0.86 | 0.16 - 4.48 | 0.86 | 1.58 | 0.71 - 3.49 | 0.26 | 1.54 | 0.18 - 13.42 | 0.70 | 1.59 | 0.39 - 6.51 | 0.52 |  |
| Orthodox Medicine | 0.99 | 0.28 - 3.43 | 0.98 | 1.53 | 0.31 - 7.53 | 0.60 | - | - | - | 1.13 | 0.13 - 9.68 | 0.91 | 1.83 | 0.46 - 7.27 | 0.39 |  |
| None | Ref. |  |  |  |  |  |  |  |  |  |  |  |  |  |  |  |
| **Symptoms experienced in the last Six months** |  |  |  |  |  |  |  |  |  |  |  |  |  |  |  |  |
| Diarrhoea | 0.78 | 0.26 - 2.34 | 0.65 | 0.64 | 0.19 - 2.17 | 0.48 | 0.71 | 0.21 - 2.39 | 0.59 | 0.59 | 0.15 - 2.23 | 0.44 | 1.49 | 0.49 - 4.47 | 0.48 |  |
| Loss of appetite | 0.60 | 0.18 - 2.02 | 0.41 | 0.56 | 0.14 - 2.28 | 0.42 | 0.31 | 0.06 - 1.50 | 0.15 | 0.58 | 0.13 - 2.66 | 0.48 | 0.93 | 0.27 - 3.23 | 0.91 |  |
| Nausea/Vomiting | 0.35 | 0.11 - 1.18 | 0.09 | 1.4 | 0.39 - 5.01 | 0.61 | 0.2 | 0.03 - 1.17 | 0.07 | 0.42 | 0.08 - 2.12 | 0.29 | 2.48 | 0.74 - 8.28 | 0.14 |  |
| Stomach pain | 0.59 | 0.20 - 1.74 | 0.20 | 0.69 | 0.21 - 2.26 | 0.54 | 0.33 | 0.09 - 1.21 | 0.09 | 0.24 | 0.05 - 1.08 | 0.06 | 1.24 | 0.42 - 3.69 | 0.71 |  |
| None | Ref. |  |  |  |  |  |  |  |  |  |  |  |  |  |  |  |
| **Source of information on parasitic infection** |  |  |  |  |  |  |  |  |  |  |  |  |  |  |  |  |
| Friends | 1.5 | 0.34 - 6.58 | 0.59 | 0.74 | 0.14 - 3.79 | 0.72 | 2.25 | 0.23 - 22.27 | 0.49 | 0.38 | 0.02 - 6.70 | 0.51 | 1.38 | 0.31 - 6.16 | 0.67 |  |
| Hospital Worker | 0.89 | 0.22 - 3.66 | 0.87 | 0.72 | 0.15 - 3.46 | 0.68 | 1.55 | 0.16 - 15.16 | 0.71 | 1.55 | 0.16 - 15.16 | 0.71 | 0.93 | 0.22 - 3.94 | 0.92 |  |
| Media | 1.37 | 0.35 - 5.42 | 0.66 | 0.85 | 0.19 - 3.84 | 0.83 | 1.66 | 0.18 - 15.31 | 0.66 | 0.88 | 0.09 - 8.87 | 0.91 | 0.75 | 0.18 - 3.08 | 0.69 |  |
| Teachers | 1.57 | 0.42 - 5.84 | 0.50 | 0.47 | 0.11 - 2.02 | 0.31 | 1.25 | 0.14 - 10.93 | 0.84 | 1.38 | 0.16 - 11.99 | 0.77 | 0.95 | 0.25 - 3.64 | 0.95 |  |
| Others | Ref. |  |  |  |  |  |  |  |  |  |  |  |  |  |  |  |

**Statistically significant: p-value ≤0.05**

**Table 3a**

| Parasitic Infection | Prevalence | Confidence Interval |
| --- | --- | --- |
| *Ascaris lumbricoides* | 0.57 | 0.50 - 0.64 |
| *Giardia duodenalis* | 0.22 | 0.16 - 0.27 |
| *Entamoeba spp* | 0.14 | 0.10 - 0.20 |
| *Trichuris trichuira* | 0.11 | 0.07 - 0.16 |
| Hookworm | 0.39 | 0.32 - 0.46 |

**Table 3b**

|  |  |  |  |
| --- | --- | --- | --- |
| ***Ascaris lumbricoides*** | **Adjusted Odds Ratio** | **95% CI** | **P-value** |
| **Source_of_food at school** |  |  |  |
| From friends | 0.74 | 0.20 – 2.70 | 0.65 |
| Home cooked food | 0.49 | 0.27 – 0.90 | 0.02** |
| Buy from street/School | Ref. |  |  |
| **Type_of_toilet** |  |  |  |
| Open defaecation | 0.43 | 0.17 – 1.09 | 0.08 |
| Pit Latrine | 0.54 | 0.25 – 1.19 | 0.13 |
| WC | 0.40 | 0.18 – 0.91 | 0.03** |
| KVIP | Ref. |  |  |

| ***Giardia duodenalis*** | **Adjusted Odds Ratio** | **95% CI** | **P-value** |
| --- | --- | --- | --- |
| **Gender** |  |  |  |
| Male | 2.20 | 0.99 – 4.85 | 0.05** |
| Female | Ref. |  |  |
| **Biting fingernails bite** |  |  |  |
| Sometimes | 0.55 | 0.26 – 1.14 | 0.11 |
| Yes | 0.28 | 0.09 – 0.84 | 0.02** |
| No | Ref. |  |  |
|  |  |  |  |

|  |  |  |  |
| --- | --- | --- | --- |
| **Entamoeba spp.** | **Odds Ratio** | **95% CI** | **P-value** |
| **Awareness of intestinal** |  |  |  |
| Yes | 0.37 | 0.15 – 0.86 | 0.02** |
| No | Ref. |  |  |
| **Parents occupation** |  |  |  |
| Civil Servant | 0.23 | 0.04 – 1.39 | 0.11 |
| Farmer | 0.17 | 0.04 – 0.78 | 0.02** |
| Trader | 0.33 | 0.07 – 1.55 | 0.16 |
| Unemployed | Ref. |  |  |

*Statistically Significant **; p-value ≤0.05*
